# Supplementary material for: Microbial Composition on Abandoned and Reclaimed Mining Sites in the Komi Republic (North Russia)
Source: Microorganisms. 2023 Mar 10;11(3):720. doi: 10.3390/microorganisms11030720 (PMC10052540; doi:10.3390/microorganisms11030720)
Supplement: Supplementary file 1 [file microorganisms-11-00720-s001.zip › microorganisms-2227093-supplementary.pdf]

Supplementary Table S1. Nutrition parameters of disturbed and reference soils.

Group	BioRepeat	TOC	pH	P	K	N-ammonium	N-nitrate
Quarry Clay	A	1.45	6.8	42	369	98.6	15.2
Quarry Clay	B	1.56	7	7.3	337	44.3	1.59
Quarry Clay	C	1.21	6.9	36	885	93	12.4
Primary Forest	A	0.66	6.4	46	59	7.62	2.5
Primary Forest	B	0.87	5.9	52	188	10.3	2.24
Primary Forest	C	0.55	5.5	67	296	19.2	0.77
Dumped Limestone	A	1.22	7.3	4.7	785	3.76	0.73
Dumped Limestone	B	1.34	7.5	6.8	118	9.36	1.38
Dumped Limestone	C	1.12	7.4	2.1	240	4.26	4.65
Secondary Forest	A	1.45	7.3	3.4	205	13.4	10.6
Secondary Forest	B	1.65	7.2	1.3	188	17.5	1.08
Secondary Forest	C	1.45	7.2	3.4	181	15.7	1.08
Reclamated Limestone	A	1.65	7.2	0.9	77	19	8.52
Reclamated Limestone	B	1.66	7.1	4.7	146	44.3	7.86
Reclamated Limestone	C	1.7	7.1	3.4	216	37.9	5.24
